# Supplementary material for: Dynamics of Mycobacterium tuberculosis Lineages in Oman, 2009 to 2018
Source: Pathogens. 2022 May 4;11(5):541. doi: 10.3390/pathogens11050541 (PMC9148118; doi:10.3390/pathogens11050541)
Supplement: Supplementary file 1 [file pathogens-11-00541-s001.zip › pathogens-1679902-supplementary.pdf]

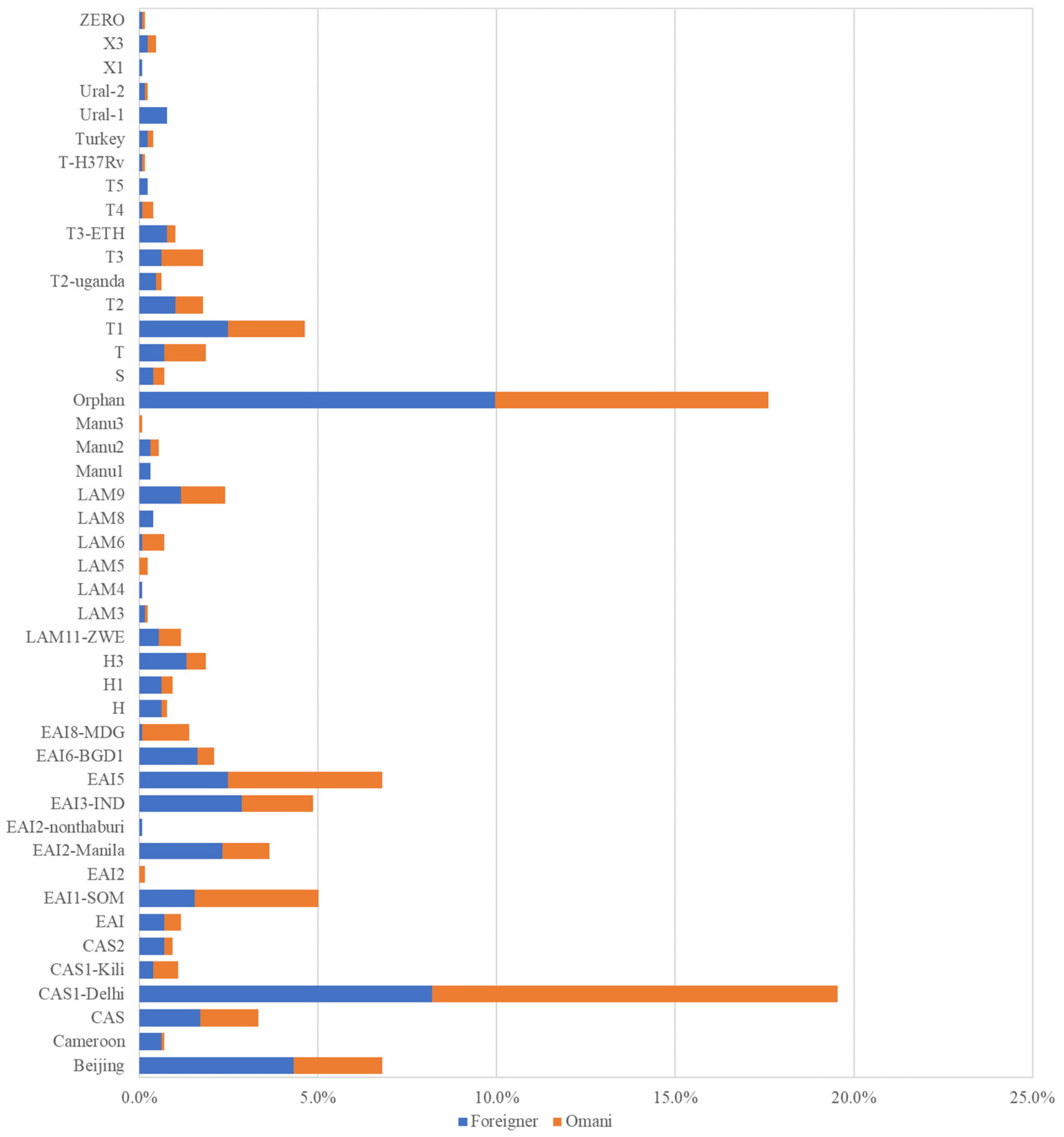

Supplementary Figure S1. Proportion of *M. tuberculosis* spoligotypes clades among Omanis and foreigners between 2009 and 2018.

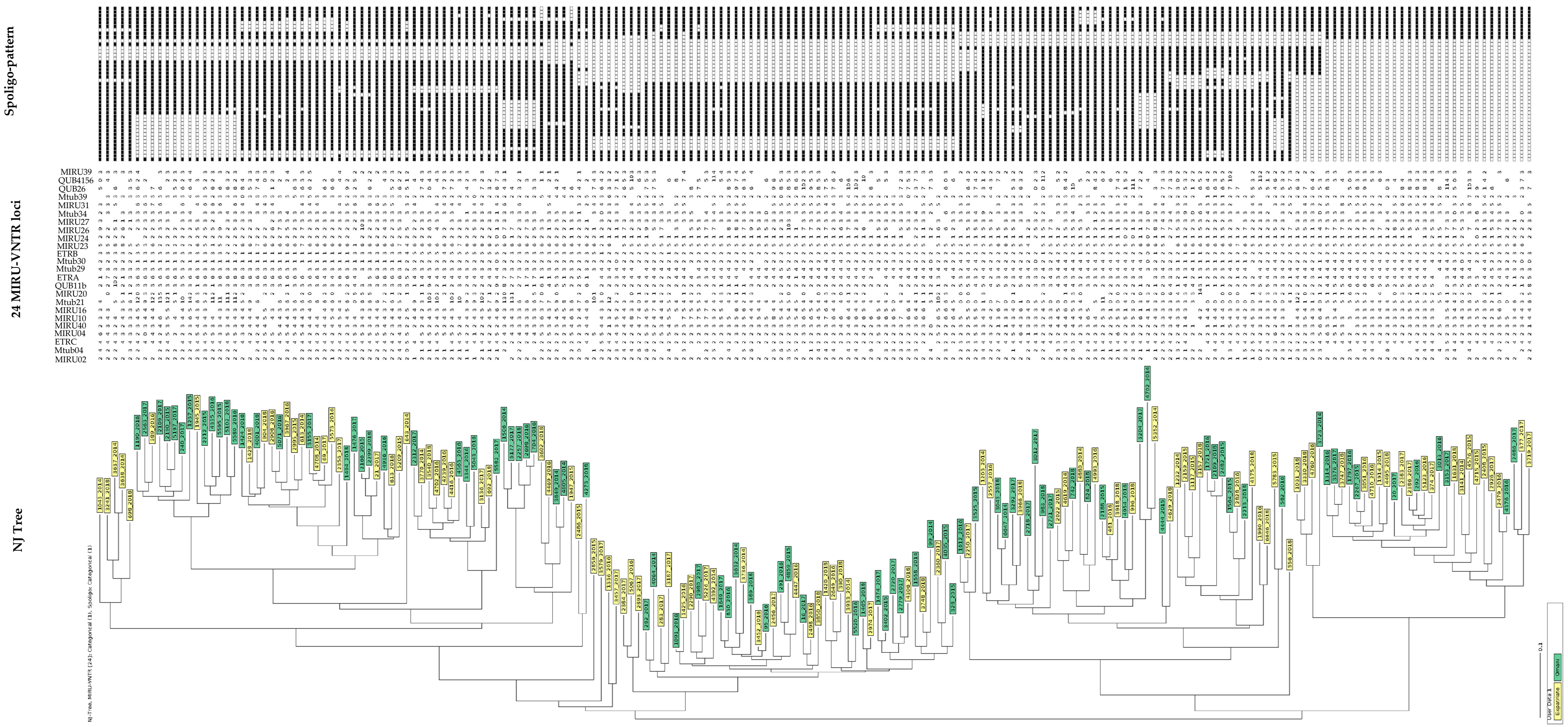

Supplementary Figure S2. Neighbor-joining (NJ) tree showing the phylogenetic relationship of 192 M. tuberculosis isolates in Oman. The NJ tree was constructed using spoligotyping and 24-loci MIRU-VNTR data. MIRU-VNTR alleles and spoligo-patterns are also represented along with the NJ tree. Isolate label was colored according to nationality, green color represents isolates collected from Omanis and yellow from foreigners

Supplementary Table S1. Sociodemographic characteristics of 2,539 TB patients from whom M. tuberculosis isolates were collected between 2009 and 2018.

	2009	2010	2011	2012	2013	2014	2015	2016	2017	2018	Total
Expatriate	79	80	90	122	138	164	143	148	116	118	1198
	31.9%	35.1%	32.6%	42.4%	50.5%	57.7%	54.8%	55.6%	51.1%	62.8%	47.2%
Omani	169	148	186	166	135	120	118	118	111	70	1341
	68.1%	64.9%	67.4%	57.6%	49.5%	42.3%	45.2%	44.4%	48.9%	37.2%	52.8%
Total	248	228	276	288	273	284	261	266	227	188	2539
Female	77	94	112	136	104	104	111	98	92	62	990
	31.0%	41.2%	40.6%	47.2%	38.1%	36.6%	42.5%	36.8%	40.5%	33.0%	39.0%
Male	171	134	164	152	169	180	150	168	135	126	1549
	69.0%	58.8%	59.4%	52.8%	61.9%	63.4%	57.5%	63.2%	59.5%	67.0%	61.0%
Pulmonary	197	171	234	245	232	235	221	214	198	155	2102
	79.4%	75.0%	84.4%	85.1%	85.0%	82.7%	84.7%	80.5%	87.2%	82.4%	82.8%
Extra-pulmonary	51	57	42	43	41	49	40	52	29	33	437
	20.6%	25.0%	15.2%	14.9%	15.0%	17.3%	15.3%	19.5%	12.8%	17.6%	17.2%
0-4	3	2	0	6	2	1	0	2	1	0	17
	1.2%	0.9%	0.0%	2.1%	0.7%	0.4%	0.0%	0.8%	0.4%	0.0%	0.7%
5-14	5	0	4	2	3	1	2	0	4	1	22
	2.0%	0.0%	1.5%	0.7%	1.1%	0.4%	0.8%	0.0%	1.8%	0.5%	0.9%
15-24	52	46	44	41	45	42	48	32	28	28	406
	21.1%	20.5%	16.1%	14.4%	16.7%	14.9%	18.6%	12.0%	12.4%	14.9%	16.1%
25-34	70	66	88	93	93	104	96	96	71	60	837
	28.3%	29.5%	32.1%	32.7%	34.6%	36.9%	37.2%	36.1%	31.4%	31.9%	33.2%
35-44	39	34	42	54	45	46	36	52	38	45	431
	15.8%	15.2%	15.3%	19.0%	16.7%	16.3%	14.0%	19.5%	16.8%	23.9%	17.1%
45-54	25	32	39	27	25	38	28	29	33	21	297
	10.1%	14.3%	14.2%	9.5%	9.3%	13.5%	10.9%	10.9%	14.6%	11.2%	11.8%
55-64	28	12	27	28	28	27	18	22	18	14	222
	11.3%	5.4%	9.9%	9.9%	10.4%	9.6%	7.0%	8.3%	8.0%	7.4%	8.8%
65+	25	32	30	33	28	23	30	33	33	19	286
	10.1%	14.3%	10.9%	11.6%	10.4%	8.2%	11.6%	12.4%	14.6%	10.1%	11.4%
Al Batinah	52	52	50	56	50	70	78	67	58	29	562
	21.7%	26.0%	22.7%	23.3%	18.3%	25.8%	31.1%	29.5%	28.6%	17.3%	24.5%
Al Buraimi	5	4	5	6	6	10	4	4	10	3	57
	2.1%	2.0%	2.3%	2.5%	2.2%	3.7%	1.6%	1.8%	4.9%	1.8%	2.5%
Al Dakhiliyah	15	12	10	8	10	34	22	16	12	7	146
	6.3%	6.0%	4.5%	3.3%	3.7%	12.5%	8.8%	7.0%	5.9%	4.2%	6.4%
Al Dhahira	3	4	6	9	4	8	13	6	12	10	75
	1.3%	2.0%	2.7%	3.8%	1.5%	3.0%	5.2%	2.6%	5.9%	6.0%	3.3%
Al Sharqiyah	9	9	9	17	17	17	18	23	17	13	149
	3.8%	4.5%	4.1%	7.1%	6.2%	6.3%	7.2%	10.1%	8.4%	7.7%	6.5%
Al Wousta	0	2	3	0	0	3	1	5	2	3	19
	0.0%	1.0%	1.4%	0.0%	0.0%	1.1%	0.4%	2.2%	1.0%	1.8%	0.8%
Dhofar	32	27	37	38	35	34	29	23	29	23	307
	13.3%	13.5%	16.8%	15.8%	12.8%	12.5%	11.6%	10.1%	14.3%	13.7%	13.4%
Musandam	2	1	2	1	3	0	0	0	0	0	9
	0.8%	0.5%	0.9%	0.4%	1.1%	0.0%	0.0%	0.0%	0.0%	0.0%	0.4%
Muscat	122	89	98	105	148	95	86	83	63	80	969
	50.8%	44.5%	44.5%	43.8%	54.2%	35.1%	34.3%	36.6%	31.0%	47.6%	42.3%

Supplementary Table S2. TB cases per 100,000 population in different provinces in Oman between 2009 and 2018.

Province	2009	2010	2011	2012	2013	2014	2015	2016	2017	2018	*P*-value*
Dhofar	10.4	10.8	11.7	11.0	9.5	9.3	7.5	5.7	6.3	5.1	0.000
Muscat	13.0	11.5	9.8	9.6	12.8	7.8	6.7	5.8	4.4	5.5	0.001
Musandam	4.9	3.2	6.4	2.9	7.7	0	0	0	0	0	0.021
Al Buraimi	4.2	5.5	6.4	6.7	7.2	10.0	3.9	3.6	8.7	2.6	0.805
Al Wusta	0.0	4.7	9.8	0.0	0.0	7.3	4.9	11.5	4.4	6.3	0.271
Al Batinah	0.0	6.7	6.1	6.2	5.3	5.8	4.5	6.0	5.0	1.7	0.969
Adh Dhahira	1.8	2.6	3.8	5.3	2.2	4.3	6.8	2.9	5.6	4.5	0.110
Ad Dakhliyah	4.5	3.7	2.9	2.2	2.6	8.4	5.3	3.8	2.6	1.5	0.700
Ash Sharqiyah	0.0	2.6	2.2	3.7	3.5	2.4	2.7	4.1	3.2	1.8	0.244

* Linear regression was conducted to test correlation between prevalence and years for each province.

Supplementary Table S3. Distribution of M. tuberculosis lineages in different provinces in Oman.

	Al Batinah	Al Buraimi	Ad Dakhiliyah	Ash Sharqiyah	Dhofar	Muscat	Total	P-value
EAI	74 (26.9%)	5 (16.1%)	15 (18.1%)	19 (21.6%)	34 (22.2%)	143 (26.2%)	288 (24.5%)	0.373
Beijing	15 (5.5%)	1 (3.2%)	5 (6.0%)	11 (12.5%)	18 (11.8%)	26 (4.8%)	76 (6.5%)	0.015
CAS	55 (20.0%)	6 (19.4%)	28 (33.7%)	19 (21.6%)	49 (32.0%)	118 (21.6%)	275 (23.4%)	0.017
T	38 (13.8%)	4 (12.9%)	10 (12.0%)	8 (9.1%)	17 (11.1%)	52 (9.5%)	129 (11.0%)	0.564
H	13 (4.7%)	2 (6.5%)	2 (2.4%)	5 (5.7%)	2 (1.3%)	24 (4.4%)	48 (4.1%)	0.300
LAM	10 (3.6%)	3 (9.7%)	3 (3.6%)	5 (5.7%)	6 (3.9%)	32 (5.9%)	59 (5.0%)	0.552
Others*	9 (3.3%)	1 (3.2%)	2 (2.4%)	6 (6.8%)	4 (2.6%)	12 (2.2%)	34 (2.9%)	0.440
Orphan	63 (22.9%)	9 (29.0%)	18 (21.7%)	15 (17.0%)	23 (15.0%)	139 (25.5%)	267 (22.7%)	0.065
Total	275	31	83	88	153	546	1176	1176
